# Caloric restriction stimulates autophagy in rat cortical neurons through neuropeptide Y and ghrelin receptors activation

**DOI:** 10.18632/aging.100996

**Published:** 2016-07-20

**Authors:** Marisa Ferreira-Marques, Célia A. Aveleira, Sara Carmo-Silva, Mariana Botelho, Luís Pereira de Almeida, Cláudia Cavadas

**Affiliations:** ^1^ CNC - Center for Neuroscience and Cell Biology, University of Coimbra, Coimbra, Portugal; ^2^ Faculty of Pharmacy, University of Coimbra, Coimbra, Portugal

**Keywords:** neuropeptide Y (NPY), ghrelin, caloric restriction, autophagy, cortical neurons

## Abstract

Caloric restriction is an anti-aging intervention known to extend lifespan in several experimental models, at least in part, by stimulating autophagy. Caloric restriction increases neuropeptide Y (NPY) in the hypothalamus and plasma ghrelin, a peripheral gut hormone that acts in hypothalamus to modulate energy homeostasis. NPY and ghrelin have been shown to be neuroprotective in different brain areas and to induce several physiological modifications similar to those induced by caloric restriction. However, the effect of NPY and ghrelin in autophagy in cortical neurons is currently not known. Using a cell culture of rat cortical neurons we investigate the involvement of NPY and ghrelin in caloric restriction-induced autophagy. We observed that a caloric restriction mimetic cell culture medium stimulates autophagy in rat cortical neurons and NPY or ghrelin receptor antagonists blocked this effect. On the other hand, exogenous NPY or ghrelin stimulate autophagy in rat cortical neurons. Moreover, NPY mediates the stimulatory effect of ghrelin on autophagy in rat cortical neurons. Since autophagy impairment occurs in aging and age-related neurodegenerative diseases, NPY and ghrelin synergistic effect on autophagy stimulation may suggest a new strategy to delay aging process.

## INTRODUCTION

Aging is an age-dependent or age-progressive decline in intrinsic physiological functions. Average human life expectancy has increased, and consequently, the prevalence of cognitive decline dementia, and neurodegenerative diseases. Aging research is now focused in finding strategies that increase both lifespan and healthspan [1].

Caloric restriction, reduction of food intake to 30-40 % below *ad libitum* intake levels without malnutrition and retaining the essential nutrients, is one of the most robust non-pharmacological interventions shown to extend median and maximum lifespan and delay the onset of age-related diseases in several species, including fruit flies, rodents and rhesus monkeys [2-11]. Caloric restriction-induced beneficial effects are mediated, at least in part, by autophagy activation [9, 12-14]. Autophagy is a degradation process of long-lived proteins and organelles and is important for cellular homeostasis maintenance [14, 15]. It is well established that the basal autophagic activity of living cells decreases with age, contributing to the different aspects of the aging phenotype and to the aggravation of detrimental age-related diseases [16, 17]. In fact, several evidences indicate that autophagy impairment is a hallmark of aging and neurodegenerative diseases [16, 18]. The beneficial roles of autophagy in nervous system are mainly associated with maintaining the normal balance between the formation and degradation of cellular proteins as defects in autophagy pathway have been linked to neurodegenerative diseases, such as Alzheimer's disease, Parkinson's disease, Huntington's disease, transmissible spongiform encephalopathy or prion disease and Machado-Joseph disease [19-28].

Caloric restriction induces a neuroendocrine response such as increasing neuropeptide Y (NPY) levels, in the arcuate nucleus of the hypothalamus [29-32]. NPY is abundantly expressed in numerous brain regions including hypothalamus, hippocampus and cerebral cortex [33]. NPY acts through G-coupled protein NPY receptors, named NPY Y_1_, Y_2_, Y_4_ or Y_5_ receptors [34]. NPY receptors activation regulates several physiological functions, such as regulation of food intake, blood pressure, body temperature, hormone and neuro-transmitters release, and modulation of pain, sexual behavior, circadian rhythms, memory processing and cognition [35]. In addition, NPY receptors activation has neuroprotective effects in different brain areas and delays neurodegenerative diseases, such as Alzheimer's, Parkinson's and Machado-Joseph disease rodent models [34, 36-38]. Recently, data obtained by our group show that caloric restriction increases NPY levels in hypothalamic neurons and NPY, per se, not only induces autophagy in hypothalamic neurons, but also mediates caloric restriction-induced autophagy, suggesting that NPY may mediate caloric restriction effects on auto-phagy [39, 40]. This effect on other brain regions, such as the cerebral cortex, was never investigated before.

Caloric restriction also increases the circulating levels of ghrelin, a peripheral orexigenic hormone synthesized predominantly in the stomach in response to fasting [41-43]. Ghrelin has a ubiquitous expression throughout the body namely in the central nervous system, in particularly in the hypothalamus and cerebral cortex [44, 45]. The actions of ghrelin are mediated through the activation of the G-coupled protein growth hormone secretagogue type 1a receptor (GHS-R1a), which also has a wide tissue distribution [43, 46]. Ghrelin is involved in the regulation of cardiovascular functions, bone metabolism and inflammation [47, 48]. Ghrelin is also involved in memory and learning and has a neuroprotective effect in neurodegenerative diseases and ischemic brain injury models [46, 48-52].

Since caloric restriction increases autophagy and both NPY and ghrelin, the aim of this study was to investigate whether NPY and ghrelin stimulates autophagy and if these peptides mediate caloric restriction-induced autophagy in rat cortical neurons. Understanding how NPY and ghrelin may act as caloric restriction mimetics by increasing autophagic clearance in cortical neurons, provides a new anti-aging mechanisms of caloric restriction that could be further explored.

## RESULTS

### Caloric restriction induces autophagy in rat cortical neurons

To investigate whether caloric restriction regulates autophagy in rat cortical cortical neurons, we monitored autophagy in rat cortical neurons exposed to a caloric restriction mimetic medium (referred as caloric restriction hereafter) by measuring the protein levels of the transient autophagosomal membrane-bound form of LC3B (LC3B-II) and sequestosome 1 (SQSTM1, also known as p62), widely used as markers of the autophagic process [53, 54].

**Figure 1:**
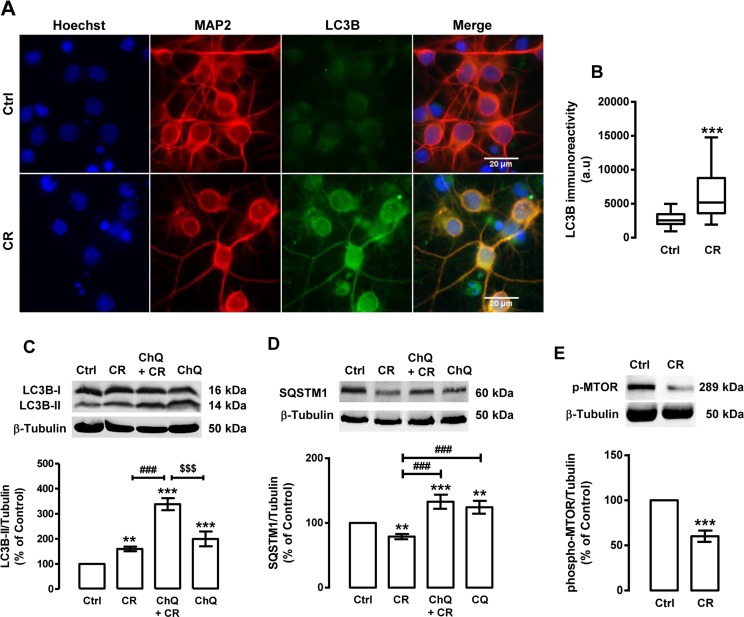
Caloric restriction increases autophagy in rat cortical neurons Primary rat cortical neurons were exposed to caloric restriction mimic medium (CR), DMEM low glucose, for 6 h. Untreated cells were used as control (Ctrl). (**A**) LC3B puncta immunoreactivity was assessed by immunocytochemistry, as described in Materials and Methods. Cells were immunolabeled for LC3B (green) and MAP2 (red). Nuclei were stained with Hoechst 33342 (blue). Figures are representative of three independents experiments. Scale bar, 20 μM. (**B**) Quantification of LC3B puncta immunoreactivity (green) *per* cell in each condition (>20 cells per group). ***p<0.001, significantly different compared to control, as determined by Student's t test. (**C**, **D** and **E**) Cells were incubated with chloroquine (ChQ, 100 μM), a lysosomal degradation inhibitor, 30 min before caloric restriction medium for 6 h. Whole cell extracts were assayed for LC3B-II (**C**), SQSTM1 (**D**), phospho-MTOR (p-MTOR) (**E**) and β-tubulin (loading control) immunoreactivity through Western blotting analysis, as described in Materials and Methods. Representative Western blots for each protein are presented above each respective graph. The results represent the mean ± SEM of, at least, five independents experiments, and are expressed as percentage of control. **p<0.01 and ***p<0.001, significantly different compared to control; ^###^p<0.001, significantly different from caloric restriction; ^$$$^p<0.001, significantly different from chloroquine-treated cells, as determined by ANOVA, followed by Bonferroni's post test.

As shown in Figure 1A and B, caloric restriction increases LC3B puncta immunoreactivity in rat cortical neurons. While untreated cells (control cells) have a diffuse LC3B cellular distribution, with few small LC3B puncta, in caloric restriction-treated cells an increase in LC3B puncta immunoreactivity was observed, suggesting an increase in autophagosome formation and autophagy induction. The levels of LC3B-II and SQSTM1 were also measured by Western blotting (Figure 1C). The results show that caloric restriction increased LC3B-II protein levels (159.9±9.1% of control) in rat cortical neurons, supporting an increase in the number of auto-phagosomes. However, an increase in LC3B-II levels and the consequent autophagosome formation does not guarantee an increase of the autophagic activity [53]. To rule out the possibility that the increase of LC3B puncta immunoreactivity was due to an inhibited auto-phagosome degradation rather than autophagosome formation, we evaluated endogenous autophagic system in the presence or absence of an inhibitor of lysosomal degradation, chloroquine [53-55]. Since LC3B-II and other autophagic substrates, as is the case of SQSTM1, are degraded at the final stages of autophagy, chloroquine treatment will impair their degradation, leading to the accumulation of both LC3B-II and SQSTM1. In the presence of chloroquine, we observed that the increase of LC3B-II induced by caloric restriction was significantly higher than in cells under caloric restriction without chloroquine (Figure 1C). Concomitant with the increase in LC3B-II steady state levels, caloric restriction decreased SQSTM1 protein content in rat cortical neurons (Figure 1D). The SQSTM1 levels were higher in cells under caloric restriction in the presence of chloroquine than in cells under caloric restriction without chloroquine (132.9±10.9% of control; Figure 1D), indicating that lysosomal degradation was inhibited. Altogether, these results show that caloric restriction increases autophagic clearance in rat cortical neurons. One of the key regulators of autophagy is the mechanistic target of rapamycin (MTOR), a conserved serine/threonine kinase that suppresses the initiation of the autophagic process when nutrients, growth factors, and energy are available [57, 58]. Inhibition of MTOR, therefore, results in activation of autophagy [57, 58]. MTOR activity can be assessed by the analysis of MTOR phosphorylation at its active site Ser^2448^. As shown in Figure 1E, caloric restriction decreased phospho-MTOR levels (60.2±6.3% of control), indicating that caloric restriction induces autophagy in rat cortical neurons through MTOR inhibition.

### Caloric restriction increases NPY and ghrelin mRNA and protein levels in rat cortical neurons

**Figure 2:**
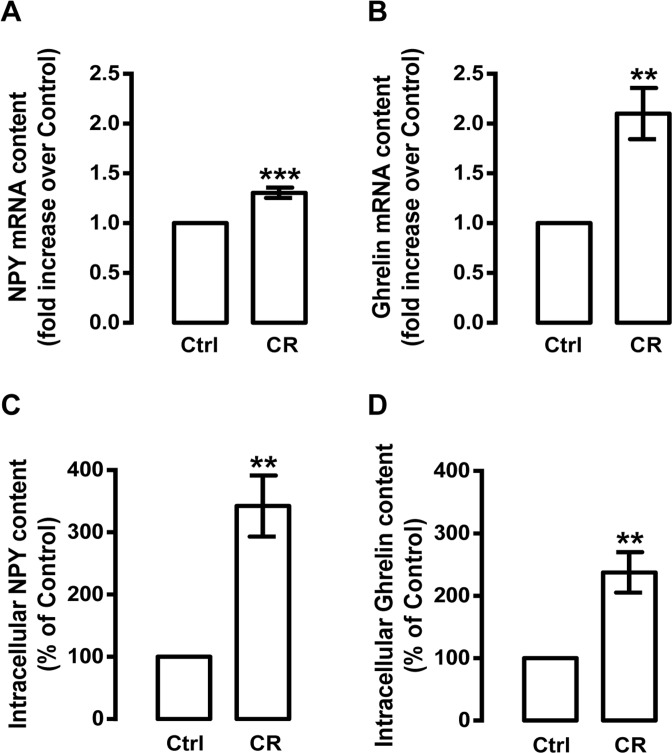
Caloric restriction increases NPY and ghrelin levels in rat cortical neurons Primary rat cortical neurons were exposed to caloric restriction medium (CR), DMEM low glucose, for 6 h. Cells in neurobasal medium with 2 % B27 supplement were used as control (Ctrl). (**A** and **B**) Total RNA was isolated and the transcript levels of NPY and ghrelin were analyzed by qRT-PCR, as described in Materials and Methods. The results represent the mean ± SEM of five independents experiments and are expressed as the relative amount compared to control. **p<0.01 and ***p<0.001, significantly different compared to control, as determined by Student's t test. (**C** and **D**) Caloric restriction increased NPY and ghrelin protein content, determined by Enzyme-Linked Immunosorbent Assays, as described in Material and Methods. The results represent the mean ± SEM of 3-4 independents experiments and are expressed as the relative amount compared to control. **p<0.01 significantly different compared to control, as determined by Student's t test.

Since caloric restriction was shown to increase the levels of hypothalamic NPY [28-31] and circulating ghrelin [39, 41, 42], we next investigated whether caloric restriction could also regulate the levels of both peptides in rat cortical neurons. As shown in Figure 2A and B, caloric restriction mimetic medium increased both NPY and ghrelin mRNA levels in primary rat cortical neurons (1.3±0.1 and 2.1±0.3 fold increase over control, respectively). Concomitantly, caloric restriction mimetic medium increased both NPY and ghrelin protein content in primary rat cortical neurons (342.3±49.0 and 237.5±32.3 percentage of control, respectively; Figure 2C and D).

### Caloric restriction stimulates autophagy through NPY receptors activation

**Figure 3:**
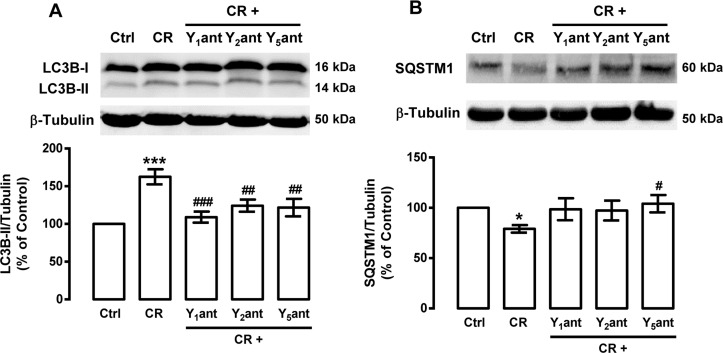
NPY receptor antagonists inhibit the stimulatory effect of caloric restriction on autophagy in rat cortical neurons Primary rat cortical neurons were incubated with NPY Y_1_ receptor antagonist BIBP3226 (Y_1_ant, 1 μM), NPY Y_2_ receptor antagonist BIIE0246 (Y_2_ant, 1 μM) or NPY Y_5_ receptor antagonist L152,800 (Y_5_ant, 1 μM), 30 min before caloric restriction medium (CR) for 6 h. Whole cell extracts were assayed for LC3B-II (**A**), SQSTM1 (**B**) and β-tubulin (loading control) immunoreactivity by Western blotting analysis, as described in Materials and Methods. Representative Western blots for each protein are presented above each respective graph. The results represent the mean ± SEM of, at least, five independents experiments, and are expressed as percentage of control. *p<0.05 and ***p<0.001, significantly different compared to control; ^#^p<0.05, ^##^p<0.01 and ^###^p<0.001, significantly different from caloric restriction, as determined by ANOVA, followed by Bonferroni's post test.

We observed that caloric restriction induces autophagy in rat cortical neurons and this is accompanied by an increase in NPY levels. Given that NPY and NPY receptors are expressed in rat cortical neurons [56], we hypothesized that NPY receptors could play a role on caloric restriction-induced autophagy in rat cortical neurons. We observed that NPY Y_1_, Y_2_ or Y_5_ receptor antagonists inhibited the stimulatory effect of caloric restriction on autophagy markers: the increase in LC3B-II (Figure 3A) and the decrease in SQSTM1 (Figure 3B) levels. These results suggest that caloric restriction-induced autophagy in rat cortical neurons is mediated by NPY Y_1_, Y_2_ or Y_5_ receptor activation.

### NPY induces autophagy in rat cortical neurons

**Figure 4:**
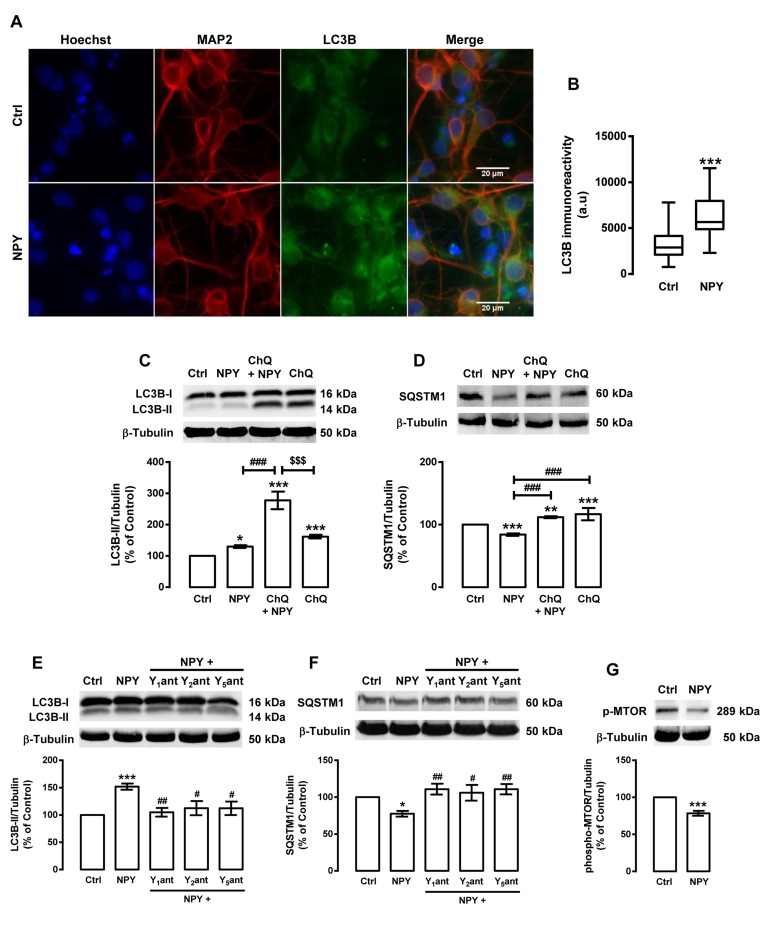
NPY increases autophagy in rat cortical neurons Primary rat cortical neurons were exposed to NPY (100 nM) for 6 h. Untreated cells were used as control (Ctrl). (**A**) LC3B distribution was assessed by immunocytochemistry assay, as described in Materials and Methods. Cells were immunolabeled for LC3B (green) and MAP2 (red, neurons). Nuclei were stained with Hoechst 33342 (blue). Figures are representative of three independents experiments. Scale bar, 20 μM. (**B**) Quantification of the number of LC3B puncta immunoreactivity (green) per cell in each condition (>20 cells per group). ***p<0.001, significantly different compared to control, as determined by Student's t test. (**C**-**G**) Cells were incubated with chloroquine (ChQ, 100 μM) (**C** and **D**), or with Y_1_ receptor antagonist BIBP3226 (Y_1_ant, 1 μM), Y_2_ receptor antagonist BIIE0246 (Y_2_ant, 1 μM) or Y_5_ receptor antagonist L152,800 (Y_5_ant, 1 μM) (**E** and **F**), 30 min before NPY (100 nM). Whole cell extracts were assayed for LC3B-II (C and E), SQSTM1 (D and F), phospho-MTOR (p-MTOR) (**G**) and β-tubulin (loading control) immunoreactivity through Western blotting analysis, as described in Materials and Methods. Representative Western blots for each protein are presented above each respective graph. The results represent the mean ± SEM of, at least, five independents experiments, and are expressed as percentage of control. *p<0.05, **p<0.01 and ***p<0.001, significantly different compared to control; ^#^p<0.05, ^##^p<0.01 and ^###^p<0.001, significantly different from NPY treatment; ^$$$^p<0.001, significantly different from chloroquine-treated cells, as determined by ANOVA, followed by Bonferroni's post test.

As the activation of NPY receptors is involved in caloric restriction-induced autophagy, we then investigated the effect of NPY *per se* on rat cortical neurons autophagy. We observed that NPY, similarly to caloric restriction, increased LC3B puncta immunoreactivity (Figure 4A and B) and LC3B-II steady state levels (129.8±4.6% of control; Figure 4C) in rat cortical neurons. Moreover, in the presence of chloroquine, the increase in LC3B-II levels was higher (277.7±28.2% of control) than in cells treated with NPY without chloroquine (Figure 4C). NPY also decreased SQSTM1 content in rat cortical neurons (Figure 4D) and this effect was inhibited in the presence of chloroquine. Moreover, we observed that NPY Y_1_, Y_2_ or Y_5_ receptor antagonists inhibited LC3B-II increase (Figure 4E) and SQSTM1 decrease (Figure 4F) induced by NPY. Overall, these results show that NPY enhances autophagic activity in rat cortical neurons, through NPY Y_1_, Y_2_ or Y_5_ receptors activation. As shown in Figure 4G, NPY decreases phospho-MTOR (Ser^2448^) levels (78.4±3.3% of control) in rat cortical neurons, which suggest that NPY, similarly to caloric restriction, induces autophagy through the inhibition of MTOR.

### Caloric restriction stimulates autophagy through ghrelin receptor activation

**Figure 5:**
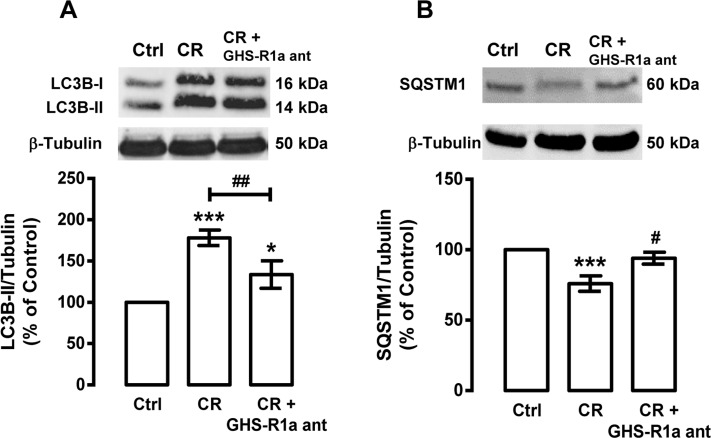
Ghrelin mediates caloric restriction-induced autophagy in rat cortical neurons Primary rat cortical neurons were treated with GHS-R1a receptor antagonist [D-Lys^3^]-GHRP-6 (GHS-R1a ant, 100 μM) 30 min before caloric restriction (CR) for 6 h. Whole cell extracts were assayed for LC3B-II (**A**), SQSTM1 (**B**) and β-tubulin (loading control) immunoreactivity through Western blotting analysis, as described in Materials and Methods. The results represent the mean ± SEM of, at least, five independents experiments, and are expressed as percentage of control. *p<0.05 and ***p<0.001, significantly different compared to control; ^#^p<0.05, ^##^p<0.01 and ^###^p<0.001, significantly different from caloric restriction, as determined by ANOVA, followed Bonferroni's post test.

Since caloric restriction increases ghrelin mRNA and protein levels in rat cortical neurons (Figure 2B), we hypothesized that ghrelin, similarly to NPY, could be involved in caloric restriction-induced autophagy in rat cortical neurons. As shown in Figure 5A and B, ghrelin receptor (GHS-R1a) antagonist ([D-Lys^3^]-GHRP-6) inhibited the increase of LC3B-II and the decrease of SQSTM1, induced by caloric restriction in rat cortical neurons. These results suggest that ghrelin receptor GHS-R1a mediates, in part, caloric restriction-induced autophagy in rat cortical neurons.

### Ghrelin induces autophagy in rat cortical neurons

**Figure 6:**
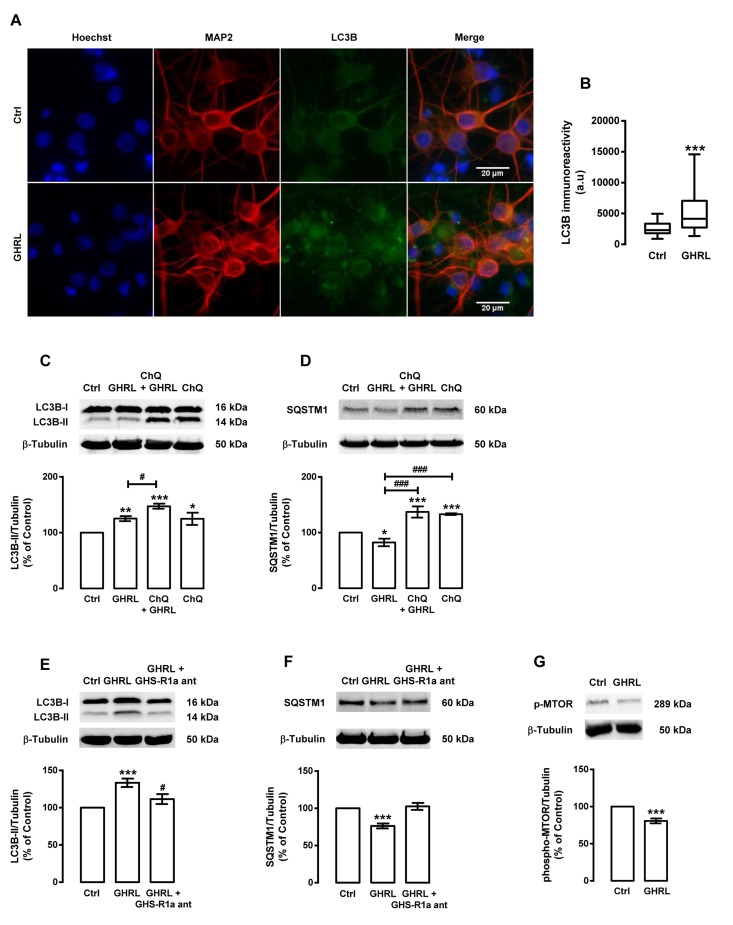
Ghrelin induces autophagy in rat cortical neurons Primary rat cortical neurons were exposed to ghrelin (GHRL, 10 nM) for 6 h. Untreated cells were used as control (Ctrl). (**A**) LC3B cellular distribution was assessed by immunocytochemistry assay, as described in Materials and Methods. Cells were immunolabeled for LC3B (green) and MAP2 (red, neurons). Nuclei were stained with Hoechst 33342 (blue). Figures are representative of three independents experiments. Scale bar, 20 μM. (**B**) Quantification of the number of LC3B puncta immunoreactivity (green) per cell in each condition (>20 cells per group). ***p<0.001, significantly different compared to control, as determined by Student's t test. (**C**-**G**) Cells were incubated with chloroquine (ChQ, 100 μM), a lysosomal degradation inhibitor (C and D) or GHS-R1a receptor antagonist [D-Lys^3^]-GHRP-6 (GHS-R1a ant, 100 μM) (**E** and **F**), 30 min before ghrelin (GHRL, 10 nM) treatment for 6 h. Whole cell extracts were assayed for LC3B-II (**C** and **E**), SQSTM1 (**D** and **F**), phospho-MTOR (p-MTOR) (**G**) and β-tubulin (loading control) immunoreactivity through Western blotting analysis, as described in Materials and Methods. Representative Western blots for each protein are presented above each respective graph. The results represent the mean ± SEM of, at least, five independents experiments, and are expressed as percentage of control. *p<0.05, **p<0.01 and ***p<0.001, significantly different compared to control; ^#^p<0.05 and ^###^p<0.001, significantly different from ghrelin treatment, as determined by ANOVA, followed Bonferroni's post test.

We next evaluated the effect of ghrelin *per se* on autophagy in rat cortical neurons. Similarly to caloric restriction and NPY, in rat cortical neurons, ghrelin induced autophagy and autophagosome formation, as shown by an increase in LC3B puncta immunoreactivity (Figure 6A), LC3B-II steady state levels (Figure 6B), and autophagic degradation, as shown by SQSTM1 protein decrease (Figure 6C and D). As expected, the GHS-R1a receptor blockage with the ghrelin receptor antagonist ([D-Lys^3^]-GHRP-6) abolished ghrelin stimulatory effects on both autophagic substrates (Figure 6E and F). Next, we observed that ghrelin decreased phospho-MTOR levels in rat cortical neurons, suggesting that ghrelin, similarly to caloric restriction and NPY, induced autophagy through the canonical inhibition of MTOR activity (Figure 6G).

### Neuropeptide Y regulates, in part, ghrelin-induced autophagy in rat cortical neurons

**Figure 7:**
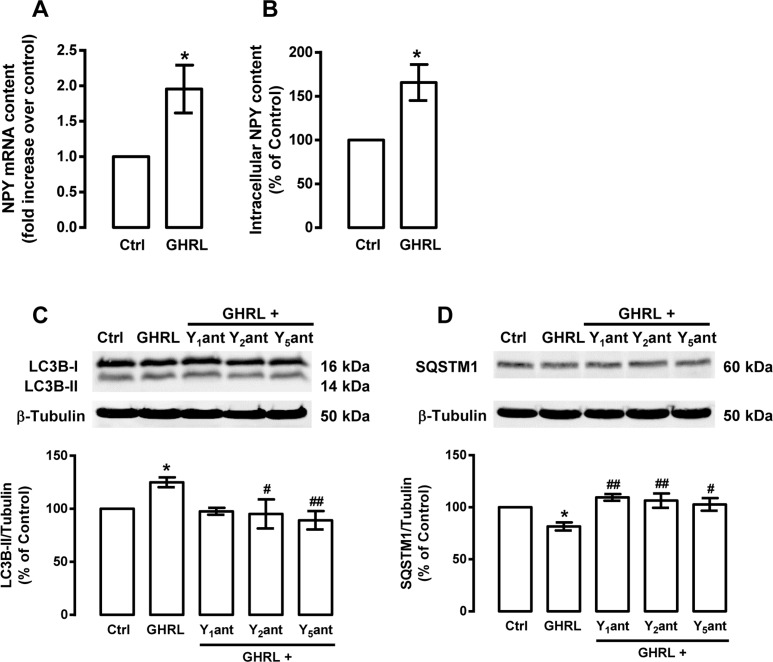
Ghrelin increases NPY content and NPY receptor antagonists block the stimulatory role of ghrelin on autophagy in rat cortical neurons Primary rat cortical neuronal cultures were exposed to ghrelin (GHRL, 10 nM) for 6 h. Untreated cells were used as control (Ctrl). (**A**) Total RNA was isolated and the transcript levels of NPY were analyzed by qPCR, as described in Materials and Methods. The results represent the mean ± SEM of five independents experiments and are expressed as the relative amount compared to control. *p<0.05, significantly different compared to control, as determined by Student's t test. (**B**) Ghrelin leads to increased NPY protein content, using an Enzyme-Linked Immunosorbent Assay, as described in Material and Methods. The results represent the mean ± SEM of three independents experiments and are expressed as the relative amount compared to control. (**C** and **D**) Cells were incubated with NPY Y_1_ receptor antagonist BIBP3226 (Y_1_ant, 1 μM), NPY Y_2_ receptor antagonist BIIE0246 (Y_2_ant, 1 μM) or NPY Y_5_ receptor antagonist L152,800 (Y_5_ant, 1 μM), 30 min before ghrelin (GHRL, 10 nM) treatment for 6 h. Whole cell extracts were assayed for LC3B-II (C), SQSTM1 (**D**) and β-tubulin (loading control) immunoreactivity through Western blotting analysis, as described in Materials and Methods. Representative Western blots for each protein are presented above each respective graph. The results represent the mean ± SEM of, at least, five independents experiments, and are expressed as percentage of control. *p<0.05 significantly different compared to control; ^#^p<0.05 and ^##^p<0.01, significantly different from ghrelin treatment, as determined by ANOVA, followed by Bonferroni's post test.

As ghrelin regulates NPY expression in hypothalamic neurons [57], we hypothesized that ghrelin could also regulate NPY levels in rat cortical neurons. As shown in Figure 7A and B, ghrelin increased NPY mRNA (1.9±0.3 fold increase over control) and NPY protein (165.7±23.5 percentage of control) levels in rat cortical neurons. This interesting observation led us to hypothesize whether NPY receptors activation through endogenous NPY could play a role on ghrelin-induced autophagy in rat cortical neurons. We observed that NPY Y_1_, Y_2_ or Y_5_ receptor antagonists significantly decreased autophagy (LC3B-II increase and SQSTM1 decrease) induced by ghrelin (Figure 7C and D). These results suggest that ghrelin enhances autophagy in rat cortical neurons, at least partially, by increasing NPY levels and consequently NPY receptors activation.

## DISCUSSION

In the present study, we show, for the first time, that NPY and ghrelin mediate autophagy stimulation induced by caloric restriction in rat cortical neurons.

These results are in agreement to recent studies that show autophagy induction in primary cortical neurons upon caloric restriction and in rodent cortical neurons upon short-term food restriction [58, 59].

Caloric restriction increases NPY in the hypothalamic neurons and herein we show that caloric restriction also increases NPY levels also in rat cortical neurons [29, 39]. In addition, we observed that NPY Y_1_, Y_2_ and Y_5_ receptor antagonists decreased the stimulatory effect of caloric restriction on autophagy, suggesting that caloric restriction-induced autophagy is dependent on NPY Y_1_, Y_2_ or Y_5_ receptor activation in rat cortical neurons. Accordingly, we recently showed that caloric restriction stimulates autophagy in rodent hypothalamic neurons and the NPY Y_1_, Y_2_ or Y_5_ receptors antagonists inhibits this stimulatory effect of caloric restriction in autophagy [39].

In the present study we showed that exogenous NPY enhances autophagy in rat cortical neurons through NPY Y_1_, Y_2_ or Y_5_ receptor activation. The similarity between the effects of caloric restriction and NPY on autophagy in cortical neuronal suggests that NPY mediates caloric restriction-induced autophagy and may be considered as a caloric restriction mimetic, as suggested by others [29, 60]. NPY and caloric restriction induce similar physiological effects, such as: hyperphagia, decreased blood glucose levels, reduced core body temperature and reduced fertility [29]. In addition, it has been shown that NPY mediates the antitumorigenic effect of caloric restriction and that caloric restriction does not increase lifespan of NPY KO mice, enlightening NPY role as a lifespan and aging regulator [61, 62]. In fact, in humans, increased NPY levels may also be correlated with lifespan benefits, since long-lived female centenarians have higher NPY plasma levels compared to younger women [63].

Aging is associated with attenuated ghrelin signaling [64, 65]. During aging, caloric restriction produces health benefits accompanied by enhanced ghrelin and ghrelin receptor (GHS-R1a) levels [41-43, 66-68]. For the first time, we show that ghrelin levels rise in rat cortical neurons upon caloric restriction, and blocking ghrelin receptor (GHS-R1a), the stimulatory effect of caloric restriction on autophagy was partially inhibited. These results suggest that ghrelin signaling may represent one of the mechanisms activated by caloric restriction. Moreover, similarly to caloric restriction, exogenous ghrelin stimulates autophagy in rat cortical neurons, by GHS-R1a receptor activation. These results suggest the potential role of ghrelin as a caloric restriction mimetic. In fact, like caloric restriction, ghrelin has several beneficial effects of age-related diseases. Ghrelin is involved in the regulation of cardiovascular functions (increase of cardiac output, decrease blood flow, protection against cardiac damage, anti-apoptotic effects), bone metabolism (increase osteoblast differentiation and bone mineral density) and inflammation (suppressing the production of cytokines) [48]. Ghrelin is also involved in memory and learning and has a neuroprotective effect in neurodegenerative diseases and ischemic brain injury models [46, 48, 69]. Indeed, ghrelin is effective in improving cell survival, reducing infarct size and rescuing memory in these animal models. Although it has been proposed that dysfunction of ghrelin signaling, through GHS-R1a ablation, may be beneficial to age-related obesity and insulin resistance [70], it has been reported that ghrelin administration in rodents and humans can possibly reverse certain characteristics of aging [71-78]. In fact, a recent study show that increasing ghrelin signalling ameliorated several age-related disorders and prolonged survival in several animal models of human aging, supporting endogenous ghrelin signalling as an important role in preventing aging-related diseases and premature death [78]. Ghrelin and GHS-R1a functions are diverse and the inter-action between their central and peripheral effects are complex, raising some controversy regarding ghrelin physiological versus pharmacological action [79]. The effectiveness of ghrelin in these roles may be impaired as ghrelin levels decrease with age, perhaps contributing to other age-related conditions like insulin resistance and diabetes, reduced fertility, and decreased performance on cognitive and memory tasks with advancing age [80, 81]. In addition, ghrelin is already being used in several clinical trials as a therapeutic strategy for the treatment of cachexia in chronic heart failure, cancer, end stage- renal disease or cystic fibrosis, frailty in elderly, anorexia nervosa, growth hormone deficient patients and sleep-wake regulation (e.g. major depression) [82, 83].

The significant overlap between caloric restriction- and ghrelin-induced physiological processes suggest that ghrelin may play a role in the beneficial effects of caloric restriction on health and lifespan. In fact, we observed that ghrelin increases NPY expression in rat cortical neurons and NPY Y_1_, Y_2_ or Y_5_ subtypes receptors antagonists inhibited the stimulatory effect of ghrelin on autophagy. These observations suggest that, similarly to caloric restriction, NPY also mediates ghrelin-induced autophagy in rat cortical neurons. The contribution of NPY in ghrelin effects has been shown by other on feeding behavior, energy balance, growth hormone secretion and gastrointestinal motility [45, 57, 84-87].

Cellular metabolic stress underpins the development of pathological conditions, of which the prevalence increases dramatically with age. In fact, a decline of NPY and ghrelin plasma levels in humans correlates with the increase of age [88-90] and the modulation of these peptides has been shown to provide neuroprotection in several neurodegenerative diseases [46, 91-94].

Overall, the present study shows that NPY and ghrelin stimulate autophagy and mediate autophagy stimulation induced by caloric restriction in rat cortical neurons. Furthermore, NPY mediates, in part, ghrelin-induced autophagy, which suggests that both peptides have a synergist effect on autophagy in rat cortical neurons. Since caloric restriction increases both NPY and ghrelin levels in cortical neurons, modulation of both peptides may be considered as a protective mechanism against impaired cortical neuronal dysfunction. Moreover, given the difficulty to implement a caloric restriction regimen, and the fact that autophagy impairment occurs in aging and age-related neurodegenerative diseases, NPY and ghrelin synergistic effect on autophagy stimulation may suggest a new approach for the improvement of both healthspan and lifespan in aging societies.

## MATERIALS AND METHODS

### Animals

Female Wistar rats were purchased from Charles River Laboratories (L'Arbresle, France). All experimental procedures were performed in accordance to the guidelines of the European Community directive for the use of animals in laboratory (2010/63/EU) translated to the Portuguese law in 2013 (Decreto-lei 113/2013). The researchers received adequate training (Felasa-certified course) and certification to perform the experiments from the Portuguese authorities (Direcção Geral de Veterinária). The present study is included in a project approved and financed by the Portuguese Science Foundation that approved the animal experimentation described. CNC – Center for Neuroscience and Cell Biology – University of Coimbra animal experimentation board approved the utilization of animals for this project (reference PTDC/SAU-FCF/099082/2008).

### Primary rat cortical neuronal cultures

Primary rat cortical neuronal cultures were obtained from cortical tissue was dissected from rats at embryonic days 18-19 (E18-19) from female rats of 16-24 weeks of age and 250-300 g of body weight. The pregnant females were sacrificed by cervical dislocation and subjected to caesarean section in order to remove the uterine horns containing the embryos. The brains were removed from the skull and cortices were dissected and meninges thoroughly removed. The cells were chemically dissociated in the presence of 0.25 % trypsin (Invitrogen) and 50 μg.mL^−1^ DNAse I (Sigma-Aldrich). After the cells were isolated, they were resuspended in high glucose (4.5 g.L^−1^) Neurobasal medium with 500 μM L-Glutamine, 2 % B27 supplement, 100 U.mL^−1^ penicillin and 100 μg.mL^−1^ streptomycin (all from Gibco), with no growth factors, and plated at a density of 1.32×10^5^ cells.cm^−2^ on poly-D-Lysine coated cell culture plates. Cortical neurons were maintained at 37°C in a humidified incubator with 5 % CO_2_/air for 8 days and the medium was replaced every fourth day by aspirating half of the medium from each well and replacing it with fresh medium.

### Experimental conditions

Cells were exposed to nutrient deprivation to mimic a caloric restriction condition, Dulbecco's Modified Eagle Medium (DMEM; Sigma-Aldrich, St. Louis, MO, USA) low glucose medium (1 g.L^−1^ glucose, 100 U.mL^−1^ penicillin and 100 μg.mL^−1^ streptomycin, without B27 supplementation), NPY (100 nM; Phoenix Europe GmbH, Karlsruhe, Germany) or acylated ghrelin (10 nM; Bachem, Bubendorf, Switzerland). Cells were also exposed to the lysosomal protein degradation inhibitor, chloroquine (100 μM; Sigma-Aldrich), NPY receptors selective antagonists (all at 1 μM; all from Bachem, Bubendorf, Switzerland; NPY Y_1_ antagonist (BIBP3226), NPY Y_2_ antagonist (BIIE0246) and NPY Y_5_ antagonist (L-152,804)), and/or ghrelin receptor antagonist ([D-Lys^3^]-GHRP-6) (100 μM; Tocris Bioscience, Bristol, UK), added to the cell culture medium 30 min prior to caloric restriction, NPY or ghrelin treatment for 6 h.

### Isolation of total RNA and cDNA synthesis

Total RNA was isolated using the RNeasy Mini Kit (Qiagen) according to the manufacturer's instructions. Briefly, cells were lysed, the total RNA was adsorbed to a silica matrix, washed with the recommended buffers and eluted with 30 μL of RNase-free water by centrifugation. Total RNA amount was quantified by optical density (OD) measurements using a ND-1000 Nanodrop Spectrophotometer (Thermo Scientific), and the purity was evaluated by measuring the ratio of OD at 260 and 280 nm. RNA samples were treated with RNase-free DNAse (Qiagen) to eliminate any contamination with genomic DNA. Reverse transcription into cDNA was carried out using the iScript Select cDNA Synthesis Kit (Bio-Rad) following the manufacturer's instructions. Briefly, 1 μg of total RNA from each sample was reverse transcribed into cDNA in a 20 μL reaction containing 1x reaction buffer, 1x random primers, and 50 units of reverse transcriptase. Reverse transcription reactions were performed in a thermocycler at 25°C for 5 min, 42°C for 30 min, 85°C for 5 min, and 4°C for 5 min. cDNA samples were then stored at −20°C until use.

### Quantitative real-time polymerase chain reaction (qPCR)

Quantitative real-time PCR was performed in an iQ5 thermocycler (Bio-Rad) using 96-well microtiter plates and the QuantiTect SYBR Green PCR Master Mix (Qiagen). The primers for the target rat genes (NPY, NM-012614), (Ghrelin, NM-021669) and the reference gene (rat HPRT, NM-012583) were pre-designed and validated by QIAGEN (QuantiTect Primers, Qiagen). A master mix was prepared for each primer set, containing the appropriate volume of 2× QuantiTect SYBR Green PCR Master Mix and 10× QuantiTectPrimer. For each reaction, 18 μL of master mix were added to 2 μL of template cDNA. All reactions were performed in duplicate (two cDNA reactions per RNA sample) at a final volume of 20 μL per well. Negative controls were performed without RNA sample, which was substituted by water. The reactions were performed according to the manufacturer's recommendations: 95°C for 15 min, followed by 40 cycles at 94°C for 15 sec, 55°C for 30 sec and 72°C for 30 sec. The melting curve protocol started immediately after amplification. The amplification efficiency for each gene and the threshold values for threshold cycle determination (Ct) were determined automatically by the iQ_5_ Optical System Software (Bio-Rad). Relative mRNA quantification was performed using the ΔCt method for genes with the same amplification efficiency. The results are expressed as the relative amount compared to control.

### Determination of NPY and ghrelin protein content

Samples were assayed for NPY and ghrelin concentration using an NPY and ghrelin EIA kit (RayBiotech, Norcross, GA, USA), respectively, according to manufacturer instructions. Cells were lysed with Krebs buffer (132 mM NaCl; 4 mM KCl; 1.4 mM MgCl_2_; 1 mM CaCl_2_; 10 mM glucose; 10 mM Hepes, supplemented with 0.001 % Tween 20, pH 7.4). Each lysate was sonicated 6x 5 sec pulses and centrifuged at 16000×g, for 8 min, at 4°C. Each supernatant was collected to a PolySorb™ tube, containing 5 % Tween 20 and 0.03 M EDTA and was stored at −80°C, until use. The protein concentration of each sample was determined by the bicinchoninic acid (BCA) protein assay (Pierce Biotechnology).

### Western blotting

Cells were lysed on ice in RIPA buffer (50 mM Tris-HCl, pH 7.4; 150 mM NaCl; 5 mM EDTA; 1 % Triton X-100; 0.5 % deoxycholate; 0.1 % sodium dodecyl sulphate (SDS); 200 μM phenyl-methylsulphonylfluoride (PMSF); 1 mM dithiothreitol (DTT), 1 mM Na3VO4; 10 mM NaF), supplemented with complete mini protease inhibitor cocktail tablet (Roche). Lysates were incubated for 15 min at 4°C, and the insoluble material was pelleted by centrifugation for 10 min at 16,000xg and 4°C. The protein concentration of each sample was determined by the bicinchoninic acid (BCA) protein assay (Pierce Biotechnology). The samples were denaturated by adding 6x concentrated sample buffer (0.5 M Tris, 30 % glycerol, 10 % SDS, 0.6 M DTT, 0.012 % bromophenol blue) and heating for 5 min at 95°C. Samples were stored at −20°C until use. Equal amounts of total protein were loaded per lane and separated by electrophoresis in SDS-PAGE, using 8-12 % gels. The protein samples were then transferred electrophoretically in CAPS buffer (0.1 M CAPS, pH 11.0; 10 % methanol) to PVDF membranes (Millipore). After blotting, the membranes were blocked in 5 % non-fat milk in Tris-buffered saline (137 mM NaCl, 20 mM Tris–HCl, pH 7.6) containing 0.1 % Tween 20 (TBS-T) for one hour at room temperature and then incubated overnight with the primary antibodies at 4°C. The primary antibodies used (all at a dilution of 1:1000; Cell Signaling) were: rabbit polyclonal anti-LC3B, anti-SQSTM1 and anti-phospho-MTOR (Ser2448). There-after, the membranes were incubated with an alkaline phosphatase-linked secondary antibody, specific to rabbit IgG or mouse IgG in a 1:10000 dilution (GE Healthcare) Protein immunoreactive bands were visualized by chemifluorescence using the ECF substrate (GE Healthcare) in a VersaDoc Imaging System (Bio-Rad) and the optical density of the bands was quantified with the Quantity One Software (Bio-Rad). The membranes were reprobed for β-tubulin immuno-reactivity (1:10000; Sigma) for equal protein loading control.

### Immunocytochemistry

After treatments, cells were washed with PBS and then fixed with ice-cold 4 % paraformaldehyde/PBS for 15 min. Cells were permeabilized with 0.25 % TX-100/PBS for 10 min, washed in PBS and blocked for one hour blocking in 10 % goat serum/PBS. Cells were incubated with primary antibodies overnight at 4°C. After incubation, cells were washed in PBS and incubated for one hour at room temperature with the respective secondary antibodies. The nuclei were stained with Hoechst 33342 (2 μg.mL^−1^; Sigma-Aldrich) during secondary antibody incubation. The coverslips were washed in PBS and mounted on glass slides with Dako Fluorescence Mounting Medium (Dako). The primary antibodies used were: rabbit anti-LC3B (1:400; Cell Signaling) and mouse anti-MAP2 (1:500; Sigma-Aldrich). The secondary antibodies (all at a dilution 1:200; Invitrogen) used were: Alexa-Fluor 488-conjugated goat anti-rabbit IgG and Alexa-Fluor 594-conjugated goat anti-mouse IgG. Cells were analyzed on a Zeiss Axiovert fluorescence microscope (Carl Zeiss). The procedure was performed for three independent cell culture preparations. Quantification of LC3B puncta immuno-reactivity in rat cortical neurons and imaging procedures using the Fiji (Fiji is Just ImageJ) software (National Health Institute) were performed. The results are expressed as the relative amount compared with control.

### Statistical analysis

Results are expressed as mean ± standard error of the mean (SEM). Data were analyzed using one-way analysis of variance (ANOVA) followed by Bonferroni's post test, or Student's unpaired t test with two-tailed p value, as indicated in figure legends. A value of p<0.05 was considered significant. Prism 5.0 (GraphPad Software) was used for all statistical analysis.

## References

[1] Flatt T (2012). A new definition of aging?. Front Genet.

[2] Colman RJ, Anderson RM, Johnson SC, Kastman EK, Kosmatka KJ, Beasley TM, Allison DB, Cruzen C, Simmons HA, Kemnitz JW, Weindruch R (2009). Caloric restriction delays disease onset and mortality in rhesus monkeys. Science.

[3] Colman RJ, Beasley TM, Kemnitz JW, Johnson SC, Weindruch R, Anderson RM (2014). Caloric restriction reduces age-related and all-cause mortality in rhesus monkeys. Nat Commun.

[4] Fowler CG, Torre P, Kemnitz JW (2002). Effects of caloric restriction and aging on the auditory function of rhesus monkeys (Macaca mulatta): The University of Wisconsin Study. Hear Res.

[5] Mattison JA, Roth GS, Beasley TM, Tilmont EM, Handy AM, Herbert RL, Longo DL, Allison DB, Young JE, Bryant M, Barnard D, Ward WF, Qi W (2012). Impact of caloric restriction on health and survival in rhesus monkeys from the NIA study. Nature.

[6] Yamada Y, Colman RJ, Kemnitz JW, Baum ST, Anderson RM, Weindruch R, Schoeller DA (2013). Long-term calorie restriction decreases metabolic cost of movement and prevents decrease of physical activity during aging in rhesus monkeys. Exp Gerontol.

[7] Masoro EJ (2006). Dietary restriction-induced life extension: a broadly based biological phenomenon. Biogerontology.

[8] Roberts SB, Schoeller DA (2007). Human caloric restriction for retardation of aging: current approaches and preliminary data. J Nutr.

[9] Bergamini E, Cavallini G, Donati A, Gori Z (2007). The role of autophagy in aging: its essential part in the anti-aging mechanism of caloric restriction. Ann N Y Acad Sci.

[10] Fontana L, Partridge L, Longo VD (2010). Extending healthy life span--from yeast to humans. Science.

[11] Fontana L, Partridge L (2015). Promoting health and longevity through diet: from model organisms to humans. Cell.

[12] Donati A (2006). The involvement of macroautophagy in aging and anti-aging interventions. Mol Aspects Med.

[13] Bergamini E, Cavallini G, Donati A, Gori Z (2003). The anti-ageing effects of caloric restriction may involve stimulation of macroautophagy and lysosomal degradation, and can be intensified pharmacologically. Biomed Pharmacother.

[14] Hansen M, Chandra A, Mitic LL, Onken B, Driscoll M, Kenyon C (2008). A role for autophagy in the extension of lifespan by dietary restriction in C. elegans. PLoS Genet.

[15] Blagosklonny MV (2010). Linking calorie restriction to longevity through sirtuins and autophagy: any role for TOR. Cell Death Dis.

[16] Cuervo AM (2008). Autophagy and aging: keeping that old broom working. Trends Genet.

[17] Lopez-Otin C, Blasco MA, Partridge L, Serrano M, Kroemer G (2013). The hallmarks of aging. Cell.

[18] Marino G, Lopez-Otin C (2004). Autophagy: molecular mechanisms, physiological functions and relevance in human pathology. Cell Mol Life Sci.

[19] Cataldo AM, Hamilton DJ, Nixon RA (1994). Lysosomal abnormalities in degenerating neurons link neuronal compromise to senile plaque development in Alzheimer disease. Brain Res.

[20] Cataldo AM, Hamilton DJ, Barnett JL, Paskevich PA, Nixon RA (1996). Properties of the endosomal-lysosomal system in the human central nervous system: disturbances mark most neurons in populations at risk to degenerate in Alzheimer's disease. J Neurosci.

[21] Stadelmann C, Deckwerth TL, Srinivasan A, Bancher C, Bruck W, Jellinger K, Lassmann H (1999). Activation of caspase-3 in single neurons and autophagic granules of granulovacuolar degeneration in Alzheimer's disease. Evidence for apoptotic cell death. Am J Pathol.

[22] Yang DS, Kumar A, Stavrides P, Peterson J, Peterhoff CM, Pawlik M, Levy E, Cataldo AM, Nixon RA (2008). Neuronal apoptosis and autophagy cross talk in aging PS/APP mice, a model of Alzheimer's disease. Am J Pathol.

[23] Anglade P, Vyas S, Javoy-Agid F, Herrero MT, Michel PP, Marquez J, Mouatt-Prigent A, Ruberg M, Hirsch EC, Agid Y (1997). Apoptosis and autophagy in nigral neurons of patients with Parkinson's disease. Histol Histopathol.

[24] Spencer B, Potkar R, Trejo M, Rockenstein E, Patrick C, Gindi R, Adame A, Wyss-Coray T, Masliah E (2009). Beclin 1 gene transfer activates autophagy and ameliorates the neurodegenerative pathology in alpha-synuclein models of Parkinson's and Lewy body diseases. J Neurosci.

[25] Kegel KB, Kim M, Sapp E, McIntyre C, Castano JG, Aronin N, DiFiglia M (2000). Huntingtin expression stimulates endosomal-lysosomal activity, endosome tubulation, and autophagy. J Neurosci.

[26] Sapp E, Schwarz C, Chase K, Bhide PG, Young AB, Penney J, Vonsattel JP, Aronin N, DiFiglia M (1997). Huntingtin localization in brains of normal and Huntington's disease patients. Ann Neurol.

[27] Liberski PP, Sikorska B, Bratosiewicz-Wasik J, Gajdusek DC, Brown P (2004). Neuronal cell death in transmissible spongiform encephalopathies (prion diseases) revisited: from apoptosis to autophagy. Int J Biochem Cell Biol.

[28] Nascimento-Ferreira I, Santos-Ferreira T, Sousa-Ferreira L, Auregan G, Onofre I, Alves S, Dufour N, Colomer Gould VF, Koeppen A, Deglon N, Pereira de Almeida L (2011). Overexpression of the autophagic beclin-1 protein clears mutant ataxin-3 and alleviates Machado-Joseph disease. Brain.

[29] Minor RK, Chang JW, de Cabo R (2009). Hungry for life: How the arcuate nucleus and neuropeptide Y may play a critical role in mediating the benefits of calorie restriction. Mol Cell Endocrinol.

[30] Bi S, Robinson BM, Moran TH (2003). Acute food deprivation and chronic food restriction differentially affect hypothalamic NPY mRNA expression. Am J Physiol Regul Integr Comp Physiol.

[31] Brady LS, Smith MA, Gold PW, Herkenham M (1990). Altered expression of hypothalamic neuropeptide mRNAs in food-restricted and food-deprived rats. Neuroendocrinology.

[32] de Rijke CE, Hillebrand JJ, Verhagen LA, Roeling TA, Adan RA (2005). Hypothalamic neuropeptide expression following chronic food restriction in sedentary and wheel-running rats. J Mol Endocrinol.

[33] Chronwall BM, DiMaggio DA, Massari VJ, Pickel VM, Ruggiero DA, O'Donohue TL (1985). The anatomy of neuropeptide-Y-containing neurons in rat brain. Neuroscience.

[34] Silva AP, Xapelli S, Grouzmann E, Cavadas C (2005). The putative neuroprotective role of neuropeptide Y in the central nervous system. Curr Drug Targets CNS Neurol Disord.

[35] Wettstein JG, Earley B, Junien JL (1995). Central nervous system pharmacology of neuropeptide Y. Pharmacol Ther.

[36] Malva JO, Xapelli S, Baptista S, Valero J, Agasse F, Ferreira R, Silva AP (2012). Multifaces of neuropeptide Y in the brain--neuroprotection, neurogenesis and neuroinflammation. Neuropeptides.

[37] Decressac M, Barker RA (2012). Neuropeptide Y and its role in CNS disease and repair. Exp Neurol.

[38] Duarte-Neves J, Goncalves N, Cunha-Santos J, Simoes AT, den Dunnen WF, Hirai H, Kugler S, Cavadas C, Pereira de Almeida L (2015). Neuropeptide Y mitigates neuropathology and motor deficits in mouse models of Machado-Joseph disease. Hum Mol Genet.

[39] Aveleira CA, Botelho M, Carmo-Silva S, Pascoal JF, Ferreira-Marques M, Nobrega C, Cortes L, Valero J, Sousa-Ferreira L, Alvaro AR, Santana M, Kugler S, Pereira de Almeida L (2015). Neuropeptide Y stimulates autophagy in hypothalamic neurons. Proc Natl Acad Sci U S A.

[40] Aveleira CA, Botelho M, Cavadas C (2015). NPY/neuropeptide Y enhances autophagy in the hypothalamus: a mechanism to delay aging?. Autophagy.

[41] Lutter M, Sakata I, Osborne-Lawrence S, Rovinsky SA, Anderson JG, Jung S, Birnbaum S, Yanagisawa M, Elmquist JK, Nestler EJ, Zigman JM (2008). The orexigenic hormone ghrelin defends against depressive symptoms of chronic stress. Nat Neurosci.

[42] Ariyasu H, Takaya K, Tagami T, Ogawa Y, Hosoda K, Akamizu T, Suda M, Koh T, Natsui K, Toyooka S, Shirakami G, Usui T, Shimatsu A (2001). Stomach is a major source of circulating ghrelin, and feeding state determines plasma ghrelin-like immunoreactivity levels in humans. J Clin Endocrinol Metab.

[43] Kojima M, Hosoda H, Date Y, Nakazato M, Matsuo H, Kangawa K (1999). Ghrelin is a growth-hormone-releasing acylated peptide from stomach. Nature.

[44] Hou Z, Miao Y, Gao L, Pan H, Zhu S (2006). Ghrelin-containing neuron in cerebral cortex and hypothalamus linked with the DVC of brainstem in rat. Regul Pept.

[45] Cowley MA, Smith RG, Diano S, Tschop M, Pronchuk N, Grove KL, Strasburger CJ, Bidlingmaier M, Esterman M, Heiman ML, Garcia-Segura LM, Nillni EA, Mendez P (2003). The distribution and mechanism of action of ghrelin in the CNS demonstrates a novel hypothalamic circuit regulating energy homeostasis. Neuron.

[46] Ferrini F, Salio C, Lossi L, Merighi A (2009). Ghrelin in central neurons. Curr Neuropharmacol.

[47] Sato T, Nakamura Y, Shiimura Y, Ohgusu H, Kangawa K, Kojima M (2012). Structure, regulation and function of ghrelin. J Biochem.

[48] Stengel A, Tache Y (2012). Ghrelin - a pleiotropic hormone secreted from endocrine x/a-like cells of the stomach. Front Neurosci.

[49] Bayliss JA, Andrews ZB (2013). Ghrelin is neuroprotective in Parkinson's disease: molecular mechanisms of metabolic neuroprotection. Ther Adv Endocrinol Metab.

[50] Miao Y, Xia Q, Hou Z, Zheng Y, Pan H, Zhu S (2007). Ghrelin protects cortical neuron against focal ischemia/reperfusion in rats. Biochem Biophys Res Commun.

[51] Moon M, Choi JG, Nam DW, Hong HS, Choi YJ, Oh MS, Mook-Jung I (2011). Ghrelin ameliorates cognitive dysfunction and neurodegeneration in intrahippocampal amyloid-beta1-42 oligomer-injected mice. J Alzheimers Dis.

[52] Spencer SJ, Miller AA, Andrews ZB (2013). The role of ghrelin in neuroprotection after ischemic brain injury. Brain Sci.

[53] Klionsky DJ, Abdelmohsen K, Abe A, Abedin MJ, Abeliovich H, Acevedo Arozena A, Adachi H, Adams CM, Adams PD, Adeli K, Adhihetty PJ, Adler SG, Agam G (2016). Guidelines for the use and interpretation of assays for monitoring autophagy (3rd edition). Autophagy.

[54] Menzies FM, Moreau K, Puri C, Renna M, Rubinsztein DC (2012). Measurement of autophagic activity in mammalian cells. Curr Protoc Cell Biol.

[55] Mizushima N, Yoshimori T, Levine B (2010). Methods in mammalian autophagy research. Cell.

[56] Gray TS, Morley JE (1986). Neuropeptide Y: anatomical distribution and possible function in mammalian nervous system. Life Sci.

[57] Wren AM, Small CJ, Fribbens CV, Neary NM, Ward HL, Seal LJ, Ghatei MA, Bloom SR (2002). The hypothalamic mechanisms of the hypophysiotropic action of ghrelin. Neuroendocrinology.

[58] Young JE, Martinez RA, La Spada AR (2009). Nutrient deprivation induces neuronal autophagy and implicates reduced insulin signaling in neuroprotective autophagy activation. J Biol Chem.

[59] Alirezaei M, Kemball CC, Flynn CT, Wood MR, Whitton JL, Kiosses WB (2010). Short-term fasting induces profound neuronal autophagy. Autophagy.

[60] Botelho M, Cavadas C (2015). Neuropeptide Y: An Anti-Aging Player?. Trends Neurosci.

[61] Chiba T, Tamashiro Y, Park D, Kusudo T, Fujie R, Komatsu T, Kim SE, Park S, Hayashi H, Mori R, Yamashita H, Chung HY, Shimokawa I (2014). A key role for neuropeptide Y in lifespan extension and cancer suppression via dietary restriction. Sci Rep.

[62] Minor RK, Lopez M, Younts CM, Jones B, Pearson KJ, Anson RM, Dieguez C, de Cabo R (2011). The arcuate nucleus and neuropeptide Y contribute to the antitumorigenic effect of calorie restriction. Aging Cell.

[63] Baranowska B, Bik W, Baranowska-Bik A, Wolinska-Witort E, Szybinska A, Martynska L, Chmielowska M (2006). Neuroendocrine control of metabolic homeostasis in Polish centenarians. J Physiol Pharmacol.

[64] Inoue H, Sakamoto Y, Kangawa N, Kimura C, Ogata T, Fujieda K, Qian ZR, Sano T, Itakura M (2011). Analysis of expression and structure of the rat GH-secretagogue/ghrelin receptor (Ghsr) gene: roles of epigenetic modifications in transcriptional regulation. Mol Cell Endocrinol.

[65] Nass R, Farhy LS, Liu J, Pezzoli SS, Johnson ML, Gaylinn BD, Thorner MO (2014). Age-dependent decline in acyl-ghrelin concentrations and reduced association of acyl-ghrelin and growth hormone in healthy older adults. J Clin Endocrinol Metab.

[66] Guan XM, Yu H, Palyha OC, McKee KK, Feighner SD, Sirinathsinghji DJ, Smith RG, Van der Ploeg LH, Howard AD (1997). Distribution of mRNA encoding the growth hormone secretagogue receptor in brain and peripheral tissues. Brain Res Mol Brain Res.

[67] Howard AD, Feighner SD, Cully DF, Arena JP, Liberator PA, Rosenblum CI, Hamelin M, Hreniuk DL, Palyha OC, Anderson J, Paress PS, Diaz C, Chou M (1996). A receptor in pituitary and hypothalamus that functions in growth hormone release. Science.

[68] Zigman JM, Jones JE, Lee CE, Saper CB, Elmquist JK (2006). Expression of ghrelin receptor mRNA in the rat and the mouse brain. J Comp Neurol.

[69] Spencer SJ, Miller AA, Andrews ZB (2013). The Role of Ghrelin in Neuroprotection after Ischemic Brain Injury. Brain Sci.

[70] Lin L, Saha PK, Ma X, Henshaw IO, Shao L, Chang BH, Buras ED, Tong Q, Chan L, McGuinness OP, Sun Y (2011). Ablation of ghrelin receptor reduces adiposity and improves insulin sensitivity during aging by regulating fat metabolism in white and brown adipose tissues. Aging Cell.

[71] Finger BC, Dinan TG, Cryan JF (2011). Behavioral satiety sequence in a genetic mouse model of obesity: effects of ghrelin receptor ligands. Behav Pharmacol.

[72] Nakazato M, Murakami N, Date Y, Kojima M, Matsuo H, Kangawa K, Matsukura S (2001). A role for ghrelin in the central regulation of feeding. Nature.

[73] Tschop M, Smiley DL, Heiman ML (2000). Ghrelin induces adiposity in rodents. Nature.

[74] Wren AM, Seal LJ, Cohen MA, Brynes AE, Frost GS, Murphy KG, Dhillo WS, Ghatei MA, Bloom SR (2001). Ghrelin enhances appetite and increases food intake in humans. J Clin Endocrinol Metab.

[75] Wren AM, Small CJ, Abbott CR, Dhillo WS, Seal LJ, Cohen MA, Batterham RL, Taheri S, Stanley SA, Ghatei MA, Bloom SR (2001). Ghrelin causes hyperphagia and obesity in rats. Diabetes.

[76] Wren AM, Small CJ, Ward HL, Murphy KG, Dakin CL, Taheri S, Kennedy AR, Roberts GH, Morgan DG, Ghatei MA, Bloom SR (2000). The novel hypothalamic peptide ghrelin stimulates food intake and growth hormone secretion. Endocrinology.

[77] Ariyasu H, Iwakura H, Yamada G, Nakao K, Kangawa K, Akamizu T (2008). Efficacy of ghrelin as a therapeutic approach for age-related physiological changes. Endocrinology.

[78] Fujitsuka N, Asakawa A, Morinaga A, Amitani MS, Amitani H, Katsuura G, Sawada Y, Sudo Y, Uezono Y, Mochiki E, Sakata I, Sakai T, Hanazaki K (2016). Increased ghrelin signaling prolongs survival in mouse models of human aging through activation of sirtuin1. Mol Psychiatry.

[79] Al Massadi O, Lopez M, Ferno J, Dieguez C, Nogueiras R (2015). What is the real relevance of endogenous ghrelin?. Peptides.

[80] Wierup N, Yang S, McEvilly RJ, Mulder H, Sundler F (2004). Ghrelin is expressed in a novel endocrine cell type in developing rat islets and inhibits insulin secretion from INS-1 (832/13) cells. J Histochem Cytochem.

[81] Fernandez-Fernandez R, Tena-Sempere M, Aguilar E, Pinilla L (2004). Ghrelin effects on gonadotropin secretion in male and female rats. Neurosci Lett.

[82] Akamizu T, Kangawa K (2012). The physiological significance and potential clinical applications of ghrelin. Eur J Intern Med.

[83] Strasser F (2012). Clinical application of ghrelin. Curr Pharm Des.

[84] Chen HY, Trumbauer ME, Chen AS, Weingarth DT, Adams JR, Frazier EG, Shen Z, Marsh DJ, Feighner SD, Guan XM, Ye Z, Nargund RP, Smith RG (2004). Orexigenic action of peripheral ghrelin is mediated by neuropeptide Y and agouti-related protein. Endocrinology.

[85] Fujimiya M, Asakawa A, Ataka K, Chen CY, Kato I, Inui A (2010). Ghrelin, des-acyl ghrelin, and obestatin: regulatory roles on the gastrointestinal motility. Int J Pept.

[86] Gualillo O, Lago F, Gomez-Reino J, Casanueva FF, Dieguez C (2003). Ghrelin, a widespread hormone: insights into molecular and cellular regulation of its expression and mechanism of action. FEBS Lett.

[87] Horvath TL, Diano S, Sotonyi P, Heiman M, Tschop M (2001). Minireview: ghrelin and the regulation of energy balance--a hypothalamic perspective. Endocrinology.

[88] Chiodera P, Volpi R, Pilla S, Cataldo S, Coiro V (2000). Decline in circulating neuropeptide Y levels in normal elderly human subjects. Eur J Endocrinol.

[89] Rigamonti AE, Pincelli AI, Corra B, Viarengo R, Bonomo SM, Galimberti D, Scacchi M, Scarpini E, Cavagnini F, Muller EE (2002). Plasma ghrelin concentrations in elderly subjects: comparison with anorexic and obese patients. J Endocrinol.

[90] Sturm K, MacIntosh CG, Parker BA, Wishart J, Horowitz M, Chapman IM (2003). Appetite, food intake, and plasma concentrations of cholecystokinin, ghrelin, and other gastrointestinal hormones in undernourished older women and well-nourished young and older women. J Clin Endocrinol Metab.

[91] Decressac M, Pain S, Chabeauti PY, Frangeul L, Thiriet N, Herzog H, Vergote J, Chalon S, Jaber M, Gaillard A (2012). Neuroprotection by neuropeptide Y in cell and animal models of Parkinson's disease. Neurobiol Aging.

[92] Decressac M, Wright B, Tyers P, Gaillard A, Barker RA (2010). Neuropeptide Y modifies the disease course in the R6/2 transgenic model of Huntington's disease. Exp Neurol.

[93] Rose JB, Crews L, Rockenstein E, Adame A, Mante M, Hersh LB, Gage FH, Spencer B, Potkar R, Marr RA, Masliah E (2009). Neuropeptide Y fragments derived from neprilysin processing are neuroprotective in a transgenic model of Alzheimer's disease. J Neurosci.

[94] Stoyanova II (2014). Ghrelin: A link between ageing, metabolism and neurodegenerative disorders. Neurobiol Dis.

